# Regulatory Mechanisms of Phytohormones in Thiocyanate-Exposed Rice Plants: Integrating Multi-Omics Profiling with Mathematical Modeling

**DOI:** 10.3390/life15030486

**Published:** 2025-03-18

**Authors:** Yi Kang, Chengzhi Li, Xiaozhang Yu

**Affiliations:** College of Environmental Science & Engineering, Guilin University of Technology, Guilin 541004, China; 1020210405@glut.edu.cn (Y.K.); 1020190262@glut.edu.cn (C.L.)

**Keywords:** thiocyanate, phytohormones, differentially expressed genes, Total Hormonal Sensitivity Index, rice

## Abstract

Plants experience various abiotic stresses, among which pollutant stress is one of the most damaging, threatening plant productivity and survival. Thiocyanate (SCN^−^), a recalcitrant byproduct of industrial processes, poses escalating threats to agroecosystems by disrupting plant hormonal homeostasis, which is critical for stress adaptation. Here, we dissect the regulatory interplay of phytohormones in rice (*Oryza sativa* L.) under SCN^−^ stress (4.80–124.0 mg SCN/L) through integrated transcriptomic and metabolomic profiling. Quantitative hormonal assays revealed dose- and tissue-specific perturbations in phytohormone homeostasis, with shoots exhibiting higher sensitivity than roots. Transcriptomic analysis revealed that a number of differentially expressed genes (DEGs) mapped in different phytohormone pathways in SCN^−^-treated rice seedlings, and their transcript abundances are tissue-specific. To identify the phytohormones governing rice’s sensitivity to SCN^−^ stress, we developed a Total Hormonal Sensitivity Index (*THSI*) through an integrative multivariate framework, which combines Modified Variable Importance in Projection (*VIP*_(*m*)_) scores to quantify hormonal fluctuations and Total Weighted Contribution Scores (*TWCS*) at the gene-level from hormonal pathways. This study establishes a system-level understanding of how phytohormonal crosstalk mediates rice’s adaptation to SCN^−^ stress, providing biomarkers for phytoremediation strategies in contaminated paddies.

## 1. Introduction

Although minor thiocyanate (SCN^−^) can be naturally produced through the microbial degradation of cyanogenic glycosides in decomposing vegetation [1], anthropogenic sources dominate environmental contamination. Key industrial emitters include coal coking, cyanide-based metal extraction, petrochemical refining, and agrochemical manufacturing [2,3,4]. The compound’s environmental persistence stems from high water solubility and resistance to aerobic degradation, enabling widespread dispersion through soil–water systems [5]. Regulatory deficiencies in effluent management have exacerbated environmental accumulation in different matrices [6]. Therefore, it is not surprising that such bioaccumulation potential elevates ecological risks, which eventually make their entry into the food chain and pose a threat to all living organisms [7,8]. In spite of available research demonstrating SCN^−^ assimilation in plants through either the COS pathway or CNO pathway, limited enzymatic efficiency results in the accumulation of SCN^−^ in plants [9]. Tissue burdens of SCN^−^ in plants adversely affect growth and development through multiple mechanisms, including growth inhibition, oxidative stress, photosynthetic impairment, hormonal imbalance, alterations in free amino acid profiles, and the suppression of antioxidant enzyme functionality [8,10,11,12,13].

Plants have developed comprehensive metabolic networks to support their growth and maintain vital cellular functions [14]. One of the most important integral components is phytohormones, which function in multiple processes of biological and chemical reactions in their primary and secondary metabolic pathways [15,16]. Phytohormones, as central regulators of plant growth, development, and stress adaptation, orchestrate intricate regulatory networks that enable plants to adapt to diverse environmental challenges [17,18]. Most phytohormones mainly include auxins (IAA), cytokinins (CK), gibberellins (GA), abscisic acid (ABA), ethylene (ET), jasmonates (JA), salicylic acid (SA), brassinosteroids (BR) [19,20,21]. Individual hormonal pathways in plants have been extensively characterized [22,23]; for instance, IAA drives cell elongation and root development, while CK promotes cell division and shoot growth [24,25]. GA regulates stem elongation and seed germination, whereas ABA induces stomatal closure during drought [26,27,28]. ET coordinates fruit ripening and senescence, while JA and SA mediate defense against herbivores and pathogens [29,30,31,32,33]. BR enhances various stress levels of tolerance and photomorphogenesis [34,35,36]. However, current studies often focus on single phytohormones in plants, and their synergistic or antagonistic crosstalk under stress conditions remains poorly resolved. This knowledge gap is particularly pronounced in staple crops like rice, where hormonal trade-offs between yield and stress resilience require urgent characterization [21].

As a staple crop feeding billions, rice relies on hormones like IAA, CK, ABA, and JA to balance resource allocation between yield-related processes and environmental resilience [21]. Despite established links between SCN^−^ exposure and oxidative/metabolic dysfunction in plants [8,10,11,12,13], the regulatory hierarchy of phytohormones in mediating rice responses remains unclear. This knowledge gap hinders the development of strategies to mitigate SCN^−^ phytotoxicity. Here, we integrate transcriptomics and hormonal profiling with mathematical modeling to unravel the regulatory modes of phytohormones in SCN^−^-stressed rice, providing a full picture of stress adaptation and identifying hub phytohormones that coordinate growth–stress trade-offs. Thus, the specific objectives of this study include (1) mapping dose-dependent phytohormone fluctuations; (2) characterizing activated genes in hormonal pathways; (3) calculating tissue-specific *VIP*_(*m*)_ through the *VIP*/*VF* integration; (4) estimating pathway-level *TWCS* from transcriptional networks; and (5) modeling tissue-specific *THSI* via *VIP*_(*m*)_-*TWCS* convergence and identifying hub hormones. This systems-level approach elucidates the regulatory circuitry governing rice’s SCN^−^ adaptation, providing actionable targets for phytoremediation strategies that coordinate growth–stress trade-offs.

## 2. Materials and Methods

### 2.1. Plant Materials and Experiment Design

Rice (*Oryza sativa* L. XZX 45) seeds were planted in small cups filled with sandy soil and grown under controlled environmental conditions (light intensity: 20,000 lux, temperature: 25 ± 0.5 °C, humidity: 60 ± 2%). The growth medium comprised a modified ISO 8692 nutrient solution, as described by Feng et al. [37]. Following 16-day cultivation in the climate chamber, uniformly sized rice seedlings were screened for further experiments. Experimental groups received hydroponic solutions supplemented with potassium thiocyanate (KSCN, Sinopharm Chemical Reagent Co., Ltd., Shanghai, China) at 0 (control), 4.80 (low), 20.0 (medium), and 124.0 mg SCN/L (high) [11]. The three concentrations used represent three effective concentrations with the growth inhibition of rice seedlings by 10%, 20%, and 50%, respectively, as determined in our previous study [11]. Vessels were sealed with aluminum foil to prevent photodegradation and algal contamination. The trial employed a completely randomized design with three biological replicates per treatment, which were harvested 72 h post-exposure for endpoint analyses.

### 2.2. Measurements of Phytohormones

Phytohormone-targeting metabolomic assays were used to determine the concentration of 17 individual phytohormones in the roots and shoots of SCN^−^-treated rice plants. After 3 d of exposure, tissue samples were harvested from different treatments with three biological replications and grounded in liquid N. Each powdery sample (100 mg) was combined with 30 μL of isotope internal standards and 1170 μL of a chilled acetonitrile/water/formic acid solution (80:19:1, *v*/*v*/*v*). The mixture was thoroughly vortexed and then subjected to ultrasonication for 25 min at 4 °C. Next, the mixture was centrifuged at 14,000× *g* for 20 min at 4 °C. The supernatant was filtered using an Ostro 25 mg 96-well plate with the aid of a positive pressure device. Subsequently, the filtrate was collected and transferred to a 2 mL EP tube for phytohormone analysis (stored at −80 °C). Targeted phytohormone metabolic assays were conducted by Shanghai Applied Protein Technology Co., Ltd. (Shanghai, China) using ultra-performance liquid chromatography (UPLC, 1290 Infinity LC, Agilent Technologies, Santa Clara, CA, USA) coupled with a QTRAP (AB Sciex 6500+).

### 2.3. Identification of Genes Activated in Different Hormonal Pathways

Total RNA was isolated from SCN^−^-treated and control rice tissues using the Ultrapure RNA Kit (CWBio, Taizhou, China), followed by the DNase I treatment (same manufacturer) to eliminate genomic DNA contamination. RNA purification was completed using the RNeasy MinElute Kit (Qiagen, Hilden, Germany). The total RNA sample QC report is provided in Supplementary Materials File S1. Comprehensive transcriptome analysis was conducted with the Agilent 4X44K rice microarray platform. All hybridization procedures, including array washing, fluorescent staining, and slide scanning, were performed by Shanghai Biotechnology Corporation (Shanghai, China) using standardized protocols. Experimental data were processed through their proprietary bioinformatics. Differentially expressed genes (DEGs) were defined using dual criteria: the fold change threshold was less than 0.5 or greater than 2.0 (*p* < 0.05) [38].

To confirm the microarray results, RT-qPCR validation was conducted using the original RNA samples. Twenty genes were randomly selected for verification. Primer sequences and amplification details are provided in Supplementary Table S1. Reactions were performed on the 7500 system (Applied Biosystems) with SYBR Green under the following cycling conditions: 95 °C for 10 s, 58 °C for 30 s, and 72 °C for 32 s (40 cycles). Rice GAPDH (LOC_Os08g03290) served as the reference gene [8]. Gene expression levels were calculated using the 2^−ΔΔCT^ method with four technical replicates [8].

The DEGs involved in the phytohormones pathways in rice plants detected from different SCN^−^ treatments were identified by the MapMan database (https://mapman.gabipd.org/, Version 3.6.0RC1, accessed on 10 January 2025). The detailed list of DEGs mapped in hormonal pathways is provided in Supplementary Materials File S2.

### 2.4. Data Analysis

Phytohormone quantification assays were performed in triplicate biological replicates, with experimental results expressed as the mean ± SD. Inter-group comparisons between control and treated samples were evaluated using Tukey’s multiple comparison test, with statistical significance defined at *p* < 0.05.

## 3. Results

### 3.1. Concentrations of Phytohormones in SCN^−^-Treated Rice Plants

Seventeen distinct phytohormones or related intermediates were identified in SCN^−^-treated rice plants: abscisic acid (ABA), salicylic acid (SA), indole-3-acetic acid (IAA), 1-aminocyclopropanecarboxylic acid (ACC), jasmonic acid (JA), jasmonoyl-isoleucine (JA-Ile), oxo-phytodienoic acid (OPDA), isopentenyladenine (iP), isopentenyladenosine (iPR), trans-zeatin (tZ), trans-zeatin riboside (tZR), cis-zeatin (cZ), cis-zeatin riboside (cZR), gibberellin 3 (GA3), gibberellin 4 (GA4), gibberellin 7 (GA7), and typhasterol (TY). These compounds were classified into the following respective biosynthetic pathways: ACC (ET pathway); JA, JA-Ile, and OPDA (JA pathway); iP, iPR, tZ, tZR, cZ, and cZR (CK pathway); GA3, GA4, and GA7 (GA pathway); and TY (BR pathway).

**Figure 1:**
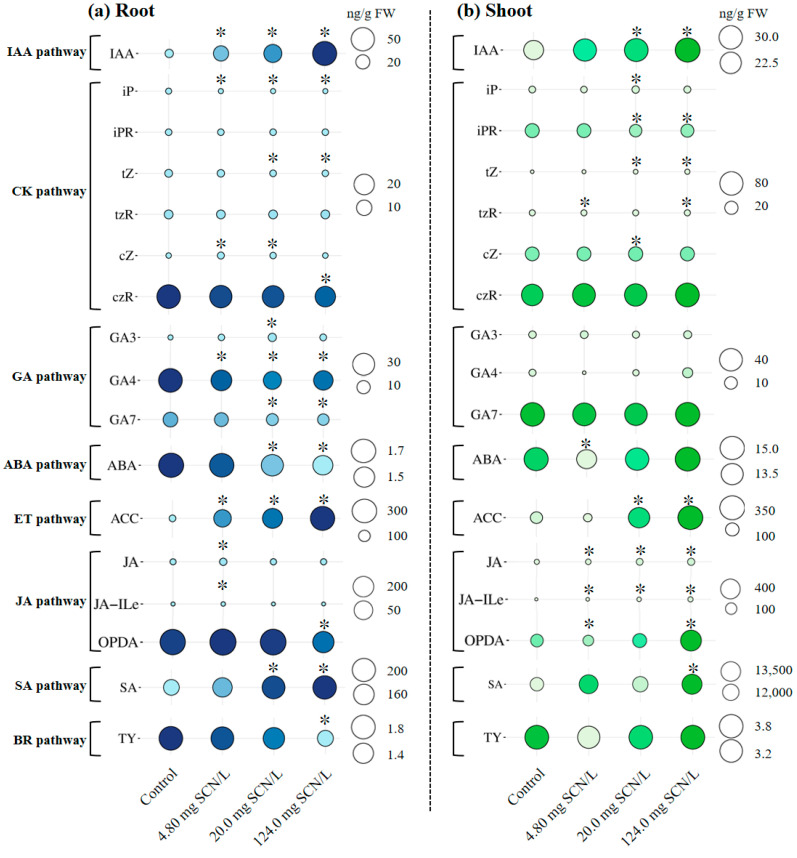
Concentrations (ng/g FW) of different phytohormones in the roots (**a**) and shoots (**b**) of rice seedlings under SCN^−^ exposure. The control is 0.0 mg Cr/L. Values are the mean of three independent biological replicates ± standard deviation. The asterisk (*) refers to the significant difference between the treatments and the control.

Figure 1 illustrates the tissue-specific concentrations of these phytohormones across different SCN^−^ treatments. Notably, the hormonal profiles exhibited both tissue-dependent variations and concentration-dependent responses to SCN^−^ exposure.

### 3.2. Identification of DEGs Activated in the Phytohormone Pathways

**Figure 2:**
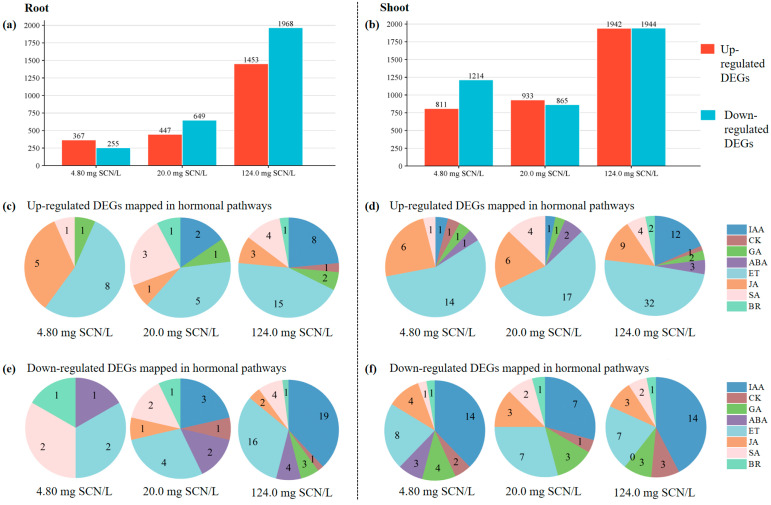
The up- and down-regulated DEGs detected in the roots (**a**) and shoots (**b**) of SCN^−^-treated rice seedlings. The number of up- and down-regulated DEGs mapped in the different hormonal pathways in the roots (**c**,**e**) and shoots (**d,f**) of rice seedlings at different SCN^−^ concentrations.

The transcriptional profiling of SCN^−^-treated rice plants was performed using the Agilent Rice Genome Array. Differential gene expression analysis revealed 2647 (622 root/2025 shoot), 2898 (1096/1798), and 7307 (3421/3886) total DEGs at SCN^−^ concentrations of 4.80, 20.0, and 124.0 mg SCN/L, respectively (Figure 2). The following tissue-specific responses were observed: in roots, 367, 447, and 1453 genes were up-regulated versus 255, 649, and 1968 down-regulated genes across the three treatments (Figure 2a). Conversely, shoots exhibited 811, 933, and 1942 up-regulated genes compared to 1214, 865, and 1944 down-regulated genes (Figure 2b).

**Figure 3:**
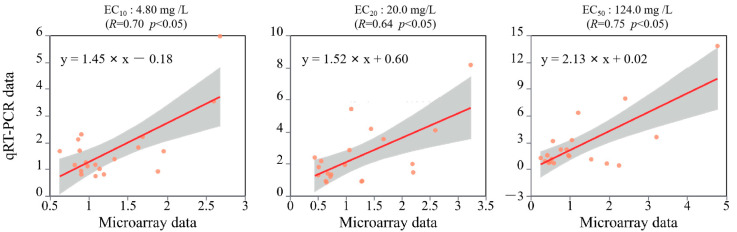
Correlation analysis of microarray data and PCR results in roots of rice seedlings at different SCN^−^ treatments.

To confirm the validity of the microarray dataset, gene expressions across SCN^−^ treatment conditions were quantified through the calculation of log2-transformed fold-changes. Subsequent qRT-PCR validation revealed concordant transcriptional profiles between both analytical platforms for all interrogated genes. The Pearson correlation coefficient (*R*) derived from the comparative analysis of expression patterns served as a critical metric for statistical validation, judged by the critical *R* for a given *n* (α = 5%) [11], demonstrating statistically significant concordance (*p* < 0.05) between the microarray and qRT-PCR quantification results (Figure 3).

To better understand the DEGs associated with the phytohormone pathways, MapMan pathway enrichment analysis was performed. The number of hormone-related DEGs from different SCN^−^ treatments is presented in Figure 2c–f. For example, 8, 5, and 15 significantly up-regulated DEGs were mapped in the ET pathway in the roots of rice plants at 4.80, 20.0, and 124.0 mg SCN/L, respectively, while 2, 4, and 16 significantly down-regulated DEGs were mapped in the ET pathway in the roots at their respective SCN^−^ treatment concentrations. In the shoots of SCN^−^-treated rice seedlings, 14, 17, and 32 significantly up-regulated DEGs were mapped in the ET pathway at 4.80, 20.0, and 124.0 mg SCN/L, respectively, while 8, 7, and 17 significantly down-regulated DEGs were mapped in the ET pathway in the roots at their respective SCN^−^ treatment concentration. We also noticed that more CK-related DEGs were identified, especially at 124.0 mg SCN/L.

### 3.3. Selection of Significantly Altered Phytohormones

To identify significantly altered phytohormones and asses their tissue-specific concentrations under SCN^−^ exposure, we implemented partial least squares–discriminant analysis (PLS-DA): a supervised multivariate method for classification and dimensionality reduction [39].

As shown in Table 1, three key validation metrics of PLS-DA, i.e., *R*^2^*_x_*, *R*^2^*_y_*, and *Q*^2^, were higher than 0.5 [40], indicating that the modeling results demonstrated high reliability.

Furthermore, phytohormones with biologically meaningful impacts were identified using the Variable Importance in the Projection (*VIP*) index, in which the *VIP* threshold was established at >1 [41]. Shoot tissues exhibited 10, 9, and 9 *VIP*-significant phytohormones at 4.80, 20.0, and 124.0 mg SCN/L, respectively, compared to 8, 19, and 11 phytohormones in the roots.

**Figure 4:**
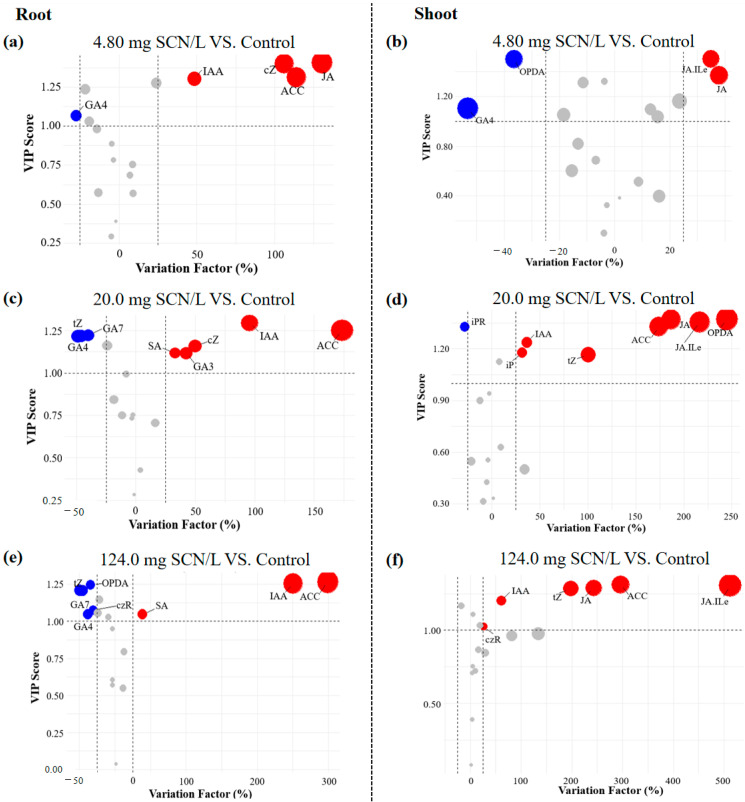
The *VIP*/*VF* integration of significantly altered phytohormones in roots ((**a**): 4.80 mg/L vs. control; (**c**): 20.0 mg/L vs. control; (**e**): 124.0 mg/L vs. control) and shoots ((**b**): 4.80 mg/L vs. control; (**d**): 20.0 mg/L vs. control; (**f**): 124.0 mg/L vs. control) of SCN^−^-treated rice seedlings. Red dots indicate the significantly positive alteration of phytohormones in responses to SCN^−^ exposure, while blue dots refer to a significantly negative alteration. Gray dots refer to no significant change.

To minimize false-positive identifications, we integrated *VIP* analysis with a variation in the factor (*VF*), which is calculated as follows:

$$VF=\frac{FC_{\left(SCN^{-}\right)}-FC_{\left(Control\right)}}{FC_{\left(Control\right)}}\times 100\%$$

where *FC*_(*SCN*)_ and *FC*_(*Control*)_ indicate the concentrations of phytohormones from the “SCN^−^” treatment and the control, respectively. The *VF* threshold was >25% or <−25% (*p* < 0.05) [42]. This dual-criterion approach revealed distinct response patterns, judged by the *VIP*_(*m*)_ values through *VIP*/*VF* integration (Figure 4). For instance, in the shoots of SCN^−^-treated rice seedlings at 4.80 mg, SCN/L, OPDA, and GA4 showed significantly negative alterations, and JA and JA-Ile positively responded to SCN^−^ exposure, wherein the roots of SCN^−^-treated rice seedlings showed a value of 4.80 mg SCN/L; GA4 showed a significantly negative alteration; and ACC, IAA, JA, and cZ responded positively to SCN^−^ exposure.

### 3.4. Estimation of the Total Weighted Contribution Score of Hormonal Pathways

MapMan pathway analysis revealed differential gene expression patterns across phytohormone pathways in SCN^−^-treated rice plants. Notably, DEGs were categorized by their mechanistic relationships with phytohormone biosynthesis: (1) the direct effect of genes encoding biosynthetic enzymes or pathway regulators, and (2) the indirect effect of genes influencing hormonal pathways through secondary mechanisms, consistent with mediation analysis principles.

Then, the total weighted contribution score of genes (*TWCS*) for each phytohormone pathway was calculated as follows:

$$TWCS_{\left(hormone\right)}=\frac{1}{n}\times \sum_{g=1}^{n}\log_{2}(FC_{g(n)})\times W_{\left(1.0\right)}+\frac{1}{m}\times \sum_{g=1}^{m}\log_{2}(FC_{g(m)})\times W_{\left(0.5\right)}$$

where *log*_2_(*FC*) refers to the expression of DEGs. *W*_(1.0)_ is the weight for direct-effect DEGs (*n*), and *W*_(0.5)_ is the weight for indirect-effect DEGs (*m*).

**Figure 5:**
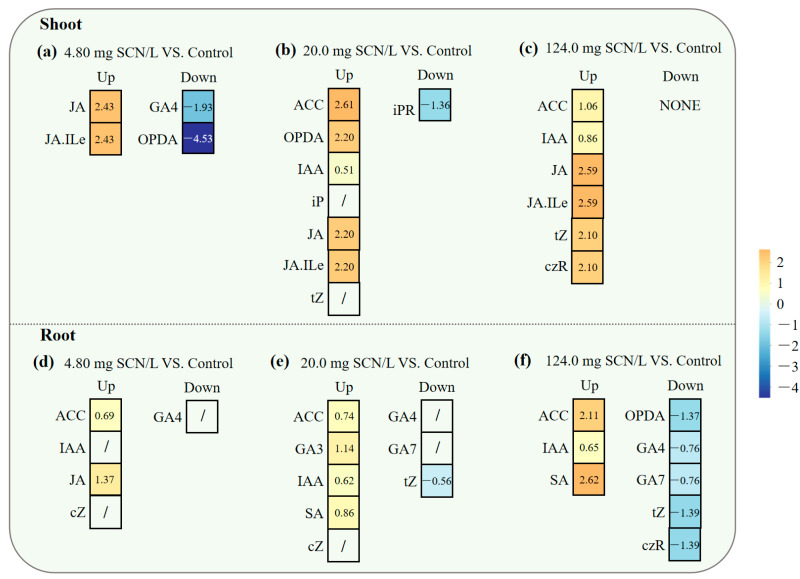
*TWCS* values quantifying pathway-specific regulatory impacts across rice tissues and different SCN^−^ concentrations.

Therefore, *TWCS* values quantifying pathway-specific regulatory impacts across SCN^−^ concentrations are summarized in Figure 5.

### 3.5. Selection of the Sensitive Phytohormones

Phytohormone concentrations in plant tissues are influenced by multifaceted regulatory mechanisms. To systematically evaluate hormonal responsiveness under SCN^−^ stress, we integrated *VIP*_(*m*)_ and *TWCS* to derive Total Hormonal Sensitivity Indices (*THSI*), calculated as follows:

$$THSI_{\left(hormone\right)}=VIP_{\left(m\right)}\times TWCS_{hormone(n)}$$

**Figure 6:**
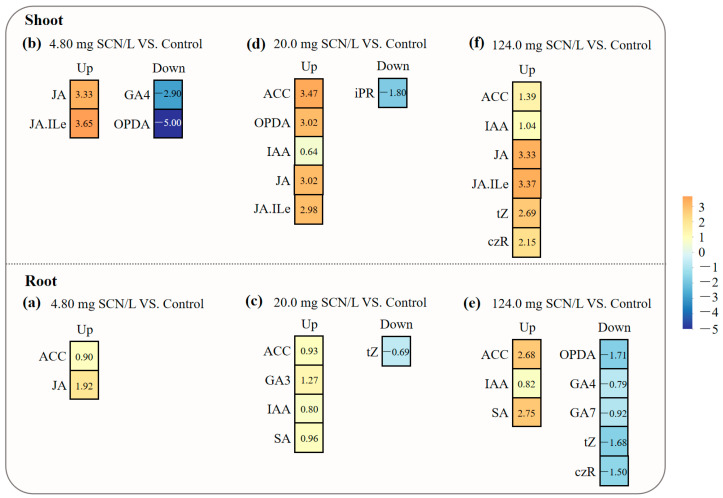
*THSI* values for phytohormones across rice tissues and different SCN^−^ concentrations.

*THSI* values for phytohormones across tissues and SCN^−^ concentrations are presented in Figure 6. We noticed that at a concentration of 4.80 mg SCN/L, JA was the most sensitive phytohormone in the roots of rice plants, followed by ACC, wherein both show a positive response to SCN^−^ stress. In the shoots of rice plants exposed to SCN^−^ at 4.80 mg SCN/L, JA-Ile showed the highest *THSI*, followed by JA, wherein both showed a positive response. At a concentration of 20.0 mg SCN/L, GA3 and ACC showed the highest *THSI* values in the roots and shoots of rice plants, respectively, demonstrating a positive response to SCN^−^ stress. At a concentration of 124.0 mg SCN/L, SA and JA-Ile had the highest *THSI* values in the roots and shoots of rice plants, respectively. Additionally, three critical patterns emerged: (1) consistently higher *THSI* values in shoots versus roots; (2) concentration-dependent sensitivity across all hormones; and (3) the tissue- and concentration-specific hierarchy of responsive phytohormones.

## 4. Discussions

Phytohormones are low-molecular-weight-signaling molecules that regulate plant growth, development, and stress adaptation [21]. Their crosstalk, particularly synergistic interactions between distinct hormonal pathways, plays a pivotal role in coordinating these processes. For example, ET regulates IAA metabolism, transport, and signaling during development; ET stabilizes the auxin efflux carrier *PIN2* in Arabidopsis roots, redistributing auxin to inhibit primary root elongation while promoting lateral root initiation [43,44]. Stepanova et al. [45] further demonstrated that ET treatment (via its precursor ACC) upregulates *YUC8* and *YUC9* expression in roots, elevating IAA levels in the elongation zone. Notably, ET signaling mutants (*ein2*, *ein3*) fail to activate YUC8/9 or accumulate auxin under ET exposure, confirming ET’s essential role in auxin biosynthesis [45]. This interplay extends to stress adaptation. In maize roots, drought-induced auxin accumulation activates ACS6 (ACC synthase 6), increasing ACC/ET levels and inhibiting root elongation, which is a water-conserving feedback mechanism [46]. Conversely, auxin-insensitive Arabidopsis mutants (*axr1*) exhibit impaired *ACS2/6* induction and reduced ET biosynthesis under drought, linking auxin signaling to osmotic stress tolerance [47]. Additionally, ARF7 stabilizes EIN3 by interacting with the EBF1/2 proteins, and auxin signaling via ARF7 suppresses EIN3 degradation, creating a feed-forward loop to amplify ET responses under stress, suggesting that the link between auxin and ethylene pathways enhances stress signal amplification [48]. In our study, ACC (ET precursor) and IAA exhibited a positive synergistic relationship in SCN^−^-stressed rice plants, except in shoots at 4.80 mg SCN/L. This tissue-specific divergence aligns with the conserved yet context-dependent nature of IAA-ET crosstalk observed across species. Also, it can be seen that *THSI* estimation yielded more positive interactions between the ET, JA, and CK pathways in rice shoots. Therefore, more comprehensive work is needed to further clarify their coordination networks and mitigate SCN^−^ toxicity.

Thiocyanate (SCN^−^), a phytotoxic pollutant, disrupts redox homeostasis by impairing antioxidant systems, thereby inducing ROS accumulation [8]. Biochemical and molecular studies further demonstrate its detrimental effects on rice physiology: SCN^−^ exposure disrupts carbon–nitrogen metabolic coordination [13] and inhibits anthocyanin transport from cytosol to vacuole in rice plants [49]. The results from Agilent 4X44K rice microarray data demonstrated that the higher concentrations of SCN^−^ stress caused more DEGs, and more DEGs were distributed in the shoots than roots, reflecting tissue-specific metabolic responses and systemic stress adaptation mechanisms. Notably, shoots, as primary photosynthetic hubs, show a marked regulation of oxidative defense and energy metabolism genes, which is consistent with their need to balance ROS mitigation with photosynthetic continuity [13,50,51]. The D1 protein serves as the core structural element of the photosystem II (PSII) reaction center in chloroplasts, exhibiting critical vulnerability to oxidative damage from ROS and ligand interactions [52,53,54]. Our data reveal that SCN^−^ exposure markedly downregulates *OspsbA* expression (the gene encoding D1) in rice shoots, indicating the SCN^−^-induced suppression of D1 biosynthesis and consequent impairment of the photosynthetic electron transport chain [12,54]. Compounding these effects, stable isotope analysis revealed root–shoot metabolic divergence under SCN^−^ stress. The molar ratio of the ^13^C to ^15^N content in roots is 0.66, suggesting efficient cyanate (CNO)/carbonyl sulfide (COS) detoxification via cyanase (CYN) and carbonyl sulfide hydrolase (COSase), while the molar ratios of the ^13^C to ^15^N content in shoots is 0.98, implying a limited enzymatic capacity for CNO/COS degradation [9]. This metabolic bottleneck explains the shoot-specific accumulation of toxic intermediates (CNO/COS) and the corresponding transcriptional burden. Collectively, these findings delineate a tissue-partitioned adaptation strategy: shoots prioritize transcriptional plasticity to preserve photosynthesis, while roots rely on metabolic efficiency for direct toxin processing [12,54].

The results from *THSI* estimation revealed the significant downregulation of GA-related genes and hormones (i.e., GA4 and GA7) under SCN^−^ exposure. This inverse correlation between GA pathway activity and stress intensity reflects an adaptive trade-off, suppressing growth metabolism to prioritize stress resilience. Consistent with this pattern, GA concentrations exhibited negative *VF* values, aligning with the dose-dependent biomass reduction in SCN^−^-treated rice seedlings [11], which is established by GA suppression mechanisms under abiotic stress [55]. The observed GA depletion corresponds to its dual role in development and stress adaptation, where tightly regulated GA levels modulate organ differentiation while maintaining stress responsiveness [56]. Mechanistically, stress-induced ROS activate SnRK2 kinases that phosphorylate DELLA proteins, stabilizing these GA signaling repressors [57]. Furthermore, the transcriptional control of GA biosynthesis genes serves as the primary regulatory node, in which DELLA proteins interface with other stress hormone pathways, enabling cross-pathway signal integration to regulate GA synthesis under stress conditions [58]. In fact, due to the involvement of different phytohormones and signaling molecules in the stress response, crosstalk among pathways is a complicated process that requires deep insights [59]. Our findings demonstrate tissue- and concentration-specific hormonal sensitivity profiles in SCN^−^-treated rice seedlings, reflecting multi-hormonal coordination through synergistic/antagonistic interactions to neutralize SCN^−^ toxicity in plants and resource allocation prioritization initiating growth–stress trade-offs mediated through hormonal signaling modulation.

## 5. Conclusions

This study employed integrated multi-omics analysis and mathematical modeling to identify the phytohormones critical for stress adaptation and regulation in rice under SCN^−^ exposure. Tissue-specific metabolomic profiling revealed distinct hormonal responses across SCN^−^ concentrations. Mapman pathway enrichment analysis highlighted the activation of the ET pathway, with more DEGs mapped. The *VIP*/*VF* integration demonstrated that significantly altered phytohormones in SCN^−^-treated rice plants are tissue- and dose-species. The *TWCS* estimation revealed pathway-specific regulatory impacts across SCN^−^ concentrations. Consistently higher *THSI* values in shoots versus roots indicated that shoots are major sites for phytohormones functioning in stress resilience, wherein positive regulation roles are chiefly associated with the ET and JA pathways. These findings systematically decode the hormonal networks coordinating stress mitigation in SCN^−^-treated rice plants, revealing strategic resource reallocation via growth–stress tradeoffs and tissue-partitioned signaling strategies for toxin management.

## Figures and Tables

**Table 1:** Key variables yielded from the PLS-DA analysis.

Comparison Groups	Variables in Shoots			Variables in Roots		
	*R* ^2^ *_x_*	*R* ^2^ *_y_*	*Q* ^2^	*R* ^2^ *_x_*	*R* ^2^ *_y_*	*Q* ^2^
SCN^−^ (4.80 mg/L) vs. Control	0.636	0.999	0.838	0.741	0.984	0.909
SCN^−^ (20.0 mg/L) vs. Control	0.635	0.995	0.874	0.761	0.997	0.921
SCN^−^ (124.0 mg/L) vs. Control	0.684	0.998	0.950	0.749	0.999	0.963

## Data Availability

The data are available in the Supplementary Materials and the public databases mentioned in this study.

## Supplementary Materials

The following supporting information can be downloaded at https://www.mdpi.com/article/10.3390/life15030486/s1. Table S1: Sequences of forward and reverse primers used in PCR analysis. File S1: Total RNA sample QC report. File S2: DEGs mapped in different hormonal pathways in rice tissues under SCN^−^ exposure.
