# Supplementary material for: Regulatory Mechanisms of Phytohormones in Thiocyanate-Exposed Rice Plants: Integrating Multi-Omics Profiling with Mathematical Modeling
Source: Life (Basel). 2025 Mar 18;15(3):486. doi: 10.3390/life15030486 (PMC11944018; doi:10.3390/life15030486)
Supplement: Supplementary file 1 [file life-15-00486-s001.zip › File S1 (Total RNA Sample QC Report) 1.pdf]

Total RNA Sample QC Report

Number of QC: 8
Sample type: rice tissues
Quant. Method: NanoDrop
QC Method: Agilent 2100 Bioanalyzer

Sample	Conc. (ng/µL)	Vol. (µL)	Total (µg)	A260/A280	RIN	28S/18S
8	188.3	50	9.42	1.93	8.6	2.0
9	211.5	200	42.30	2.02	N/A	1.0
10	230	50	11.50	1.90	9.0	2.1
11	158.1	200	31.62	2.04	N/A	1.1
12	128.8	50	6.44	1.85	N/A	1.5
13	205.8	200	41.16	2.00	N/A	1.1
14	127	200	25.40	1.99	N/A	1.0
15	158	200	31.60	2.01	N/A	1.1

Electrophoresis Image (Attachment)

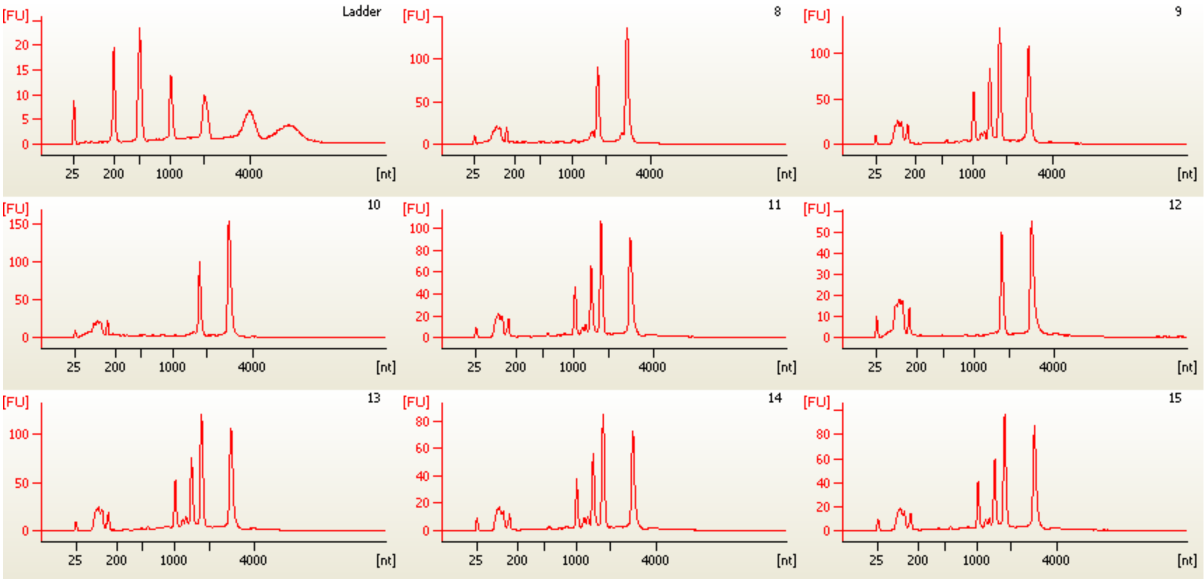

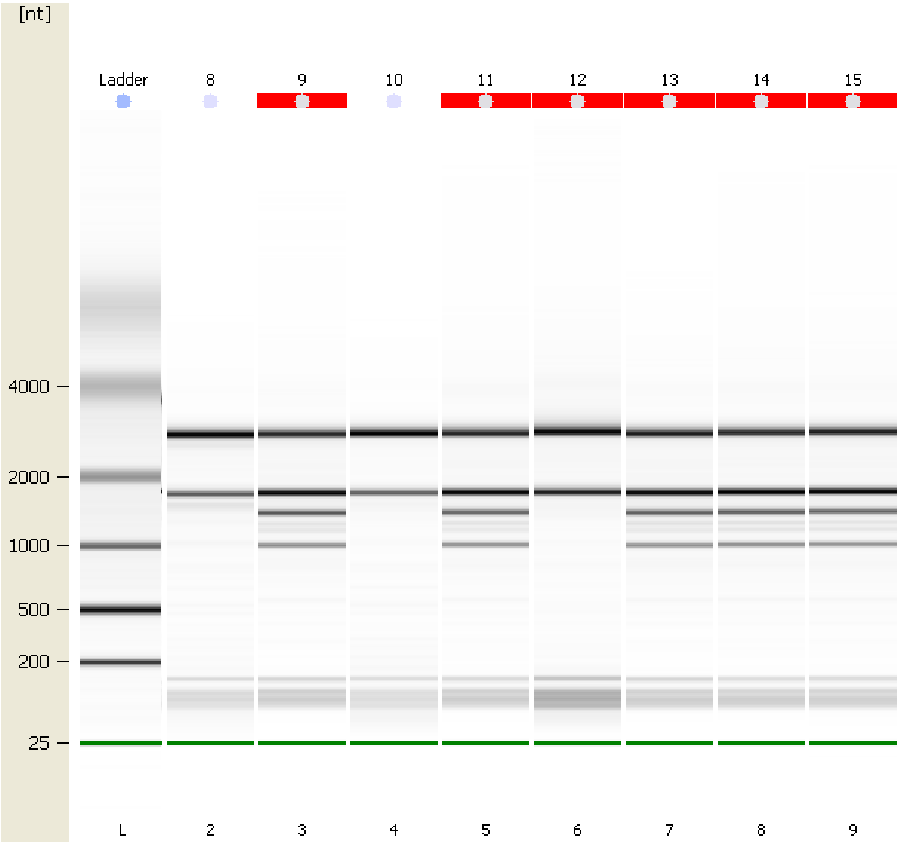

[nt]
Ladder
8
9
10
11
12
13
14
15
4000
2000
1000
500
200
25
L
2
3
4
5
6
7
8
9
