# Supplementary material for: Regulatory Mechanisms of Phytohormones in Thiocyanate-Exposed Rice Plants: Integrating Multi-Omics Profiling with Mathematical Modeling
Source: Life (Basel). 2025 Mar 18;15(3):486. doi: 10.3390/life15030486 (PMC11944018; doi:10.3390/life15030486)
Supplement: Supplementary file 1 [file life-15-00486-s001.zip › Table S1. Sequence of forward and reverse primers used in PCR analysis 1.pdf]

Table S1. Sequence of forward and reverse primers used in PCR analysis

Enzyme	Gene symbol	MSU ID	Primer sequences(5'-3')	Amplicon size (bp)
NADH dehydrogenase	*OsNDB2*	LOC_Os08g04630.1	F-GTAGAGGATGCCCAAAAGAT<br>R-ACAAGGTGCTGAATAGAAGG	206
	OsNDB3	LOC_Os05g26660.1	F-GAACTGGCAAGAAGAGGGTG<br>R-ACTACGTGCTTCAACTGTGC	170
ubiquinone	*OsNDUFA9*	LOC_Os02g57180.1	F-ATGTTCCTCTTCCTATTGC<br>R-ACTTCGTATCTTCTCACTG	269
Carbonic anhydrase	*OsCA*	LOC_Os04g33670.1	F-TGCCGCAAATCAATCTGGAG<br>R-GGATTAGAGCTGCCCTTGGT	205
Rhodanese	*OsSTR7*	LOC_Os12g24020.1	F-GAGGGTGATGAGGAGCGTG<br>R- GTCGTGGGCGTGGTAGAG	214
Cysteine synthesis	*OsRCS2*	LOC_Os06g36840.1	F-CGTACAGGCTCGATTGGTTG<br>R-GCCTTTCTGAACAGCAACCA	190
Lipoxygenase	*OsLOX2*	LOC_Os03g08220.1	F-AGAGCTGCCAACAAAGAGGG<br>R-TTCCGAACACCTGTGCCATC	129
	OsLOX2;1	LOC_Os08g39840.1	F-TCCCGTCCTTCTTCGTCATC<br>R-CTCTGAGCCATGCGAACTTG	189
	OsLOX2;3	LOC_Os08g39850.1	F-CAGCAAGCAGTTCCCTTACC<br>R-GCAGGTTCAGGCTCAGATTG	224
	OsLOX12	LOC_Os12g37350.1	F-TTGGAGGTTCGACATGGAGG<br>R-ATTCGTTTGCACCTCGTTCC	241
Nitrate reductase	*OsNIA1*	LOC_Os02g53130.1	F-ACTGGTGCTGGTGCTTCTGG<br>R-CGGCTGGGTGTTGAGGGACT	104
Glutamine Synthetase	*OsGS2*	LOC_Os04g56400.1	F-ATCACTTCGCCATGACTTGC<br>R-GACGTACGGGTCCATGTTTG	208
anthranilate synthase α-subunit	*OsOASA1*	LOC_Os03g61120.1	F- TTGTTGGGATGCTCTTCGAG<br>R- AAGTGCGATGTCCATGTCTC	163
Flavin monooxygenase-like enzyme	*OsYUCCA3*	LOC_Os01g53200.1	F- GTGTTCTCGTTGTTGGGAGT<br>R- GGGGATTTGCATTGTGGTTG	75
	OsYUCCA6	LOC_Os07g25540.1	F- CAGTGGCTCAAGGGAAGTG<br>R- CTGAGGAGACACCAGAGAGG	133
tryptophan aminotransferase	*OsTAR1*	LOC_Os05g07720.1	F- GCCGACTGTTCCACTTC<br>R- TCGTCCACCCCTTCCTT	187
aminocyclopropane-1-carboxylate oxidase	*OsACO3*	LOC_Os02g53180.1	F- CTGTGCGAGAATCTGGGC<br>R- GGTTGTAGAAGGAGGCGA	364
	OsACO4	LOC_Os11g08380.1	F- GGTGAAGAAGGTGTGCAGTG<br>R- GCTCCCTCCTGTACTCCATC	230
	OsACO6	LOC_Os05g05600.1	F-CGTTGTCTGAGGCTGATGGT<br>R-ATGGCGTCCAGGTCAATGAG	85
	OsACO7	LOC_Os01g39860.1	F-AAGAGCTTCGTGTACCGCC<br>R-GTCCTTGTTCAGGTGGAGGTT	90
glyceraldehyde-3-phosphate dehydrogenase	*OsGAPDH1*	LOC_Os08g03290.1	F-GACAGCAGGTCGAGCATCTTC<br>R-CAGGCGACAAGCTTGACAAAG	74
